# The Cultural Adaption of a Sobriety Support App for Alaska Native and American Indian People: Qualitative Feasibility and Acceptability Study

**DOI:** 10.2196/38894

**Published:** 2023-02-07

**Authors:** Susan Brown Trinidad, Aliassa L Shane, Tiffany R Guinn, Charlene R Apok, Ann F Collier, Jaedon P Avey, Dennis M Donovan

**Affiliations:** 1 Department of Bioethics and Humanities School of Medicine University of Washington Seattle, WA United States; 2 Southcentral Foundation Anchorage, AK United States

**Keywords:** alcohol misuse, sobriety support, peer support, smartphone app, community reinforcement approach, mobile phone

## Abstract

**Background:**

Despite high rates of alcohol abstinence, Alaska Native and American Indian (ANAI) people experience a disproportionate burden of alcohol-related morbidity and mortality. Multiple barriers to treatment exist for this population, including a lack of culturally relevant resources; limited access to or delays in receiving treatment; and privacy concerns. Many ANAI people in the state of Alaska, United States, live in sparsely populated rural areas, where treatment access and privacy concerns regarding peer-support programs may be particularly challenging. In addition, prior research demonstrates that many ANAI people prefer a self-management approach to sobriety, rather than formal treatment. Taken together, these factors suggest a potential role for a culturally adapted smartphone app to support ANAI people interested in changing their behavior regarding alcohol use.

**Objective:**

This study was the first phase of a feasibility and acceptability study of a culturally tailored version of an off-the-shelf smartphone app to aid ANAI people in managing or reducing their use of alcohol. The aim of this qualitative needs assessment was to gather insights and preferences from ANAI people and health care providers serving ANAI people to guide feature development, content selection, and cultural adaptation before a pilot test of the smartphone app with ANAI people.

**Methods:**

From October 2018 to September 2019, we conducted semistructured interviews with 24 ANAI patients aged ≥21 years and 8 providers in a tribal health care organization in south-central Alaska.

**Results:**

Participants generally endorsed the usefulness of a smartphone app for alcohol self-management. They cited anonymity, 24/7 access, peer support, and patient choice as key attributes of an app. The desired cultural adaptations included ANAI- and land-themed design elements, cultural content (eg, stories from elders), and spiritual resources. Participants considered an app especially useful for rural-dwelling ANAI people, as well as those who lack timely access to treatment services or prefer to work toward managing their alcohol use outside the clinical setting.

**Conclusions:**

This needs assessment identified key features, content, and cultural adaptations that are being implemented in the next phase of the study. In future work, we will determine the extent to which these changes can be accommodated in a commercially available app, the feasibility of implementation, and the acceptability of the culturally adapted version of the app among ANAI users.

## Introduction

### Background

Despite high levels of alcohol abstinence and advances in community-centered protective interventions, rates of alcohol-induced morbidity and mortality remain disproportionally high among Alaska Native and American Indian (ANAI) people in the United States [1-3]. Additional research aimed at addressing this disparity must be grounded in an understanding of sociohistorical and cultural contexts. In addition to enduring the intergenerational effects of the historical trauma associated with colonization, ANAI people experience continuing forms of oppression, such as structural racism and various forms of economic duress, that increase propensity to alcohol misuse [4,5].

Prior research has found that reservation-dwelling ANAI people in the Southwest and Northern Plains are more likely than non-Hispanic White people to seek and receive alcohol treatment [6,7]. However, it is unclear whether rates are similar among urban-dwelling ANAI people—the majority of Native peoples in the United States—or among those who do not live on reservations, particularly given the documented differences in alcohol consumption patterns across cultural groups and regions [8]. ANAI people report numerous barriers to alcohol treatment, including lack of transportation, stable housing, and employment, as well as believing that they should be able to handle the problem on their own, that it would improve on its own, and that others could not help [9-11]. In a recent study, ANAI participants who needed but did not seek treatment endorsed an average of 4.14 barriers, compared with 3.67 barriers among non-Hispanic White participants [10].

Technology-enabled tools, under the general heading of eHealth, are being explored as 1 approach to supporting individuals’ self-management of alcohol consumption [12]. Tools that include a community or other peer-support component may be especially useful for ANAI individuals who prefer a non–treatment-based approach [9,13]. One expert has defined eHealth as “an emerging field in the intersection of medical informatics, public health and business, referring to health services and information delivered or enhanced through the internet and related technologies” [14]. In general, IT has been demonstrated to be an effective form of health self-management in underserved populations [15-20]. A recent meta-analysis found that eHealth interventions are associated with higher treatment adherence among groups with an increased risk for adverse health outcomes, especially when multimodal content (eg, videos, games, or quizzes) is used and when the app facilitates direct communication with providers [21]. Mobile, internet-based interventions have shown promise for providing convenient, resource-efficient access to both the prevention and treatment of substance use–related problems [22-29]. A recent systematic review of the feasibility and effects of digital interventions for substance misuse reported that more complex interventions demonstrated stronger effect sizes (small to moderate) and were more likely to produce positive effects than simpler approaches [28].

Despite concerns regarding access to digital technology among ANAI people, a 2015 survey of ANAI adults in south-central Alaska reported that approximately 98% of the ANAI respondents had computer access, 97% had email access, and 94% had mobile phones [30]. Among mobile phone users, 60% had internet access through their mobile phones [30]. Although internet services may be less available in rural areas, many ANAI people living in remote settings are able to access the internet through local schools, government and tribal offices, and libraries [31,32]. Several studies have reported that Indigenous peoples are frequent users of smartphones and social media and rely on both to obtain health information and communicate with friends and family [33-36].

### Culturally Relevant Interventions

Prior research supports the idea that cultural adaptation of therapeutic interventions can promote patient engagement, increase treatment retention, and improve treatment outcomes [37,38]; yet, there are few published examples of culturally adapted practices for treatment of substance abuse with urban, rural, or reservation-based ANAI communities in general, let alone for technologically based treatments. This leaves a gap for providers in knowing what types of culturally relevant interventions to integrate into eHealth apps for sobriety support. The overall study will adapt a sobriety support app, Connections (CHESS Health), for use by ANAI people living in south-central Alaska. In the next phase of this research, we will work with patients and providers to assess the acceptability, feasibility, and measurable effects of this tool.

## Methods

### Setting

This study took place at Southcentral Foundation (SCF), an Alaska Native–owned and –operated tribal health organization based in Anchorage, Alaska. SCF provides health and related services to >65,000 ANAI people living in Anchorage, the Matanuska-Susitna Borough, and 55 outlying villages, most with <500 residents. SCF strives to deliver quality care in the context of challenging logistics (eg, vast geographic distances, extreme weather, and transportation challenges) across numerous distinct cultural and language groups. SCF also offers the Family Wellness Warriors program, which seeks to serve ANAI individuals wishing to address the spiritual, emotional, mental, and physical effects of domestic violence, abuse, and neglect [39,40].

### Ethics Approval

The study protocol was reviewed and approved by the SCF board of directors, which serves as the community advisory board for this research; the SCF research oversight committee; and the Alaska Area Institutional Review Board of the Indian Health Service (1287137-5).

### Recruitment

Eligible patients were ANAI adults aged ≥21 years, who (1) were eligible to receive health care services at SCF, (2) resided within the Anchorage Service Unit (including rural and urban communities), and (3) were interested in self-management and peer support for alcohol misuse and overuse. Neither a diagnosis of alcohol use disorder nor a history of treatment services was required for participation. Research staff confirmed eligibility verbally during the recruitment process. Oral informed consent was obtained from all participants. Study data were deidentified, coded, and maintained separately from individual identifiers. Patient participants received a US $50 gift card at the conclusion of the interview or by postal mail. Providers were not compensated for study participation.

To ensure that the sample represented both patients currently in recovery and those interested in self-management of their alcohol use, the patient sample was stratified by the presence or absence of negative outcomes associated with alcohol consumption based on a short self-report screener, the revised version of the Short Inventory of Problems (SIP-2R) [41,42]. A cutoff score of ≤7 was used to indicate absence of (or very low) drinking problems in the last 3 months and is equivalent to a percentile ranking of <10% of treatment-seeking men and women [43]. The SIP-2R was completed on paper for in-person recruitment; individuals who asked that the questions be read aloud were invited to complete the survey in a private room.

Recruitment strategies for patient enrollment included flyers displayed at SCF primary care clinics, community health clinics, behavioral health clinics, and alcohol treatment program waiting areas, as well as through social media, email, and word of mouth. Flyers described the topic of the research interviews, eligibility criteria, incentives, time commitment, and a contact telephone number. In addition, study staff used a recruitment table in the lobby of the Anchorage Native Primary Care Center and at SCF-sponsored community events and gatherings. Study staff answered questions and provided information to interested patients. Those interested were screened for eligibility, informed of study procedures, and asked for contact information to schedule an interview. For telephone enrollment, a member of the study staff administered the SIP-2R over the telephone. This approach did not yield sufficient representation of rural-dwelling patients. To address this, we worked with internal SCF stakeholders to develop social media posts that were posted on community-specific Facebook pages.

SCF providers who offer substance abuse services to ANAI people were invited to participate in the study through company email, flyers, and presentations at clinic staff meetings. Providers were purposively recruited to ensure rural and urban participation, as well as a range of clinical specialties that included physicians, physician assistants, substance abuse counselors, behavioral health providers, and administrators.

### Data Collection

We used written guides (one for patients and another for providers) to conduct semistructured interviews with participants between October 2018 and September 2019. The interviews were 1 to 2 hours long and were audio recorded, transcribed, and redacted before analysis. Topics in the patient guide included the acceptability of a smartphone app to support changes in alcohol use, how patients self-manage and use peer support for sobriety, desired features and content, the potential role of clinicians in the app, how the app could be tailored to reflect cultural values, and planned surveys. The provider interview guide paralleled the patient guide, with additional questions about how such an app could be integrated with clinical care.

### Data Analysis

We performed a descriptive thematic analysis of the transcripts [44]. Two team members (SBT and CRA) divided the transcripts and read them independently to familiarize themselves with the data. After the first reading round, they took notes and drafted potential thematic codes using a combination of deductive categories (ie, based on the interview guides) and inductive categories (ie, arising from the data). They exchanged transcripts and made additional notes and refinements to the thematic categories, reviewing patterns, finalizing themes, and selecting quotations that exemplified the themes. Throughout the analytic process, the analysts met regularly to discuss and resolve discrepancies. When coding was complete, they drafted a preliminary summary of the results, which was reviewed and discussed by the full research team before beginning work on this report.

## Results

### Overview

Participants shared their perspectives regarding the acceptability of a smartphone app for sobriety support and provided feedback about cultural adaptation as well as the utility and desirability of particular features. In the following sections, we present the participants’ verbatim comments.

### Participant Characteristics

In total, 32 participants were enrolled in the study: 24 (75%) patients and 8 (25%) providers. In keeping with tribal review requirements, limited demographic data are presented to protect the identity of participants. Patient characteristics are presented in Table 1. Because of the potential for identifying individual providers in our small sample, and in accordance with the guidance of the tribal review board, detailed demographic data are not reported for provider participants. However, 75% (6/8) of the providers interviewed were between the ages of 35 and 44 years, 63% (5/8) identified as urban, and 75% (6/8) had >5 years of experience in leadership roles at SCF.

**Table 1:** Patient participant characteristics (N=24)^a^.

Characteristics			Values, n (%)	
Sex, female			13 (54)	
**Age (years)**				
	21 to 34			6 (25)
	35 to 54			8 (12)
	≥55			10 (43)
**SIP-2R^b^ score**				
		Low (≤7)	15 (63)	
		High (≥8)	9 (37)	
**Residency**				
	Urban		17 (71)	
	Rural		7 (29)	

^a^Per tribal review requirements, cells with n≤5 are not reported.

^b^SIP-2R: Short Inventory of Problems, revised.

### Acceptability

We began the interviews with a general description of what a sobriety support smartphone app might look like and asked participants what they thought about making such a tool available to SCF patients. Patients generally endorsed the idea, with reactions ranging from excitement (using words such as “amazing” and “wonderful”) to comments that such a tool might be helpful for some individuals, including younger people and those who are comfortable using electronic technologies, even if they might not use it themselves. The ability of a smartphone app to be available any time, at the user’s discretion, and independent of formal treatment services was especially appreciated. Participants noted that apps allow users to get help right away, without waiting for a group meeting or an appointment with a counselor. They commented that apps can overcome common barriers to obtaining timely treatment and peer support, including transportation challenges related to weather or finances.

Patients endorsed the opportunity for users to engage anonymously, citing concerns about privacy, shame, and stigma associated with alcohol misuse as barriers to seeking peer support or treatment, particularly in small, rural communities. We also heard that an app could be a useful resource for people who may not be able to access other forms of peer support because of lack of transportation, free time, or childcare. Unprompted, participants cited specific features they would consider useful (such as tracking tools and lists of local resources), while noting that a key benefit of a smartphone app is that each individual user can determine which features and content best meet their needs in the moment.

Among those who expressed less enthusiasm about an app for sobriety support, we heard a range of views. Some of the participants pointed to economic and technical barriers, noting that many people do not have smartphones, lack sufficient data plans or the ability to pay for additional data, or do not possess the confidence or technical skills to make use of a complex app. Others said that face-to-face relationships play a critical role in recovery that cannot be fully replicated on the web while noting that app-mediated peer support might be beneficial for individuals who have not yet decided to actively work toward sobriety.

Providers were in favor of making an app available and echoed many of the themes we heard from patients. They underscored the importance of offering multiple modalities for individuals seeking to manage their alcohol use, particularly those who have not yet decided whether to engage with treatment services. A provider explained the importance of self-management support this way:

We struggle even in treatment providing people with skills and resources that they can generalize to their lives and use consistently. So something that would be either a reminder, or a platform where somebody can say, “I’m having a trigger...what are some ideas on how to cope with this in the moment?”

Provider 5

According to the providers, a smartphone app might have special value in rural communities, among younger patients, and for those who are uncomfortable seeking help “because of the fear of judgment and stigma around behavioral health and substance use” (Provider 6).

### Features and Functionality

Among the app features discussed in the interviews, participants most strongly endorsed those connected with finding and maintaining one’s motivation, including tools for goal setting and tracking progress, as well as reminders of one’s motivations for changing behavior around alcohol consumption; positive feedback, including positive recognition or “rewards” for reaching one’s goals; and affirmations, daily encouragements, and inspirational quotations. The ability to set personalized goals was strongly endorsed; patient participants spoke of “reasons” for sobriety as important touchstones for individuals to be able to remind themselves of why they are making the effort to manage their alcohol consumption behaviors.

Participants frequently mentioned the ability to track days sober. Patient participants said that reminders of “how far you’ve come” can be motivating, but a provider participant expressed concern that tracking days sober could be demotivating if an individual interpreted that information as evidence of failure. A participant, highlighting the holistic view of recovery and sobriety expressed by many of the patients in this study, recommended that tracking features not be limited to alcohol use:

Maybe they can track, like, today I went hunting, and being in tune with my cultural side is a good thing for my sobriety. So yeah, maybe just tracking...like, today I went to a potluck and I volunteered to get an elder her plate of food.

Patient 12

Patient participants endorsed recognition or rewards—such as animated confetti graphics or “coins” or other tokens of progress—for achieving sobriety goals. Some suggested that app-generated positive feedback be shared publicly so that others in one’s social network could offer their congratulations. We also heard that the app should include encouragement for users who are experiencing relapse to help them avoid all-or-nothing thinking, as well as a way for users to build a list of self-management strategies that have worked for them in the past; for example, a patient said that the app should help them define a plan in advance, outlining “What am I gonna do when I feel this way?” (Patient 19).

As noted previously, participants emphasized the ability of a smartphone app to provide immediate access to help at any time. Participants expected the app to include an emergency button that would link a user in crisis to emergency medical services, a crisis line, or other clinical resources. They also expressed interest in an in-app contact list that would provide instant access via telephone or SMS text message to the user’s identified help network for moral support or advice short of a medical emergency.

### Peer Support

Patient participants cited the ability to decide when and how to connect with others who are facing similar issues or those in recovery as a primary advantage of a sobriety support app; for example, a participant stated as follows:

I love the idea of a community online that people can talk to. I know that it works for other 12-step groups, phones, call-ins. There’s phone meetings. There’s online chat meetings. I like the idea of mentorship even....Maybe there’s a way for me, for example, to go get—well, I like that person. Now I wanna connect with them and put them into my little support network. So now I have my list of my five people that I communicate with regularly, and when one of them is not available I'll go to the next one. There’s no wrong way to do it. There’s a thousand right ways to do it, I guess you could say.

Patient 14

Convenience and 24/7 access to peer support were important as well; for example, a patient participant made the following observation:

[T]his gives them an opportunity to connect with people, especially at those weird times or those weird hours, you know, like 3 AM, or you’re in the middle of doing something and—it’s more immediate. You can reach out in the moment, instead of having to wait until five in the afternoon to go to a meeting or call somebody.

Patient 5

Another patient participant spoke of the value of peer support this way:

I’ve been talking about this from the mentor-mentee relationship, but there’s also lateral relationships that are just as beneficial, because...what I’ve experienced is that, when it really comes down to it, mentors may have some great wisdom, but they’re also human. They go through the same struggles, the same human struggles. So oftentimes mentees can be a mentor to their mentor just by nature of the fact that one person is loving on another person and relating to another person.

Patient 14

Both patients and providers noted that the peer-support component might be especially useful for individuals in rural communities, as in this comment from a patient:

In the village some people don’t have—you know, don’t really trust too many people, and it’s hard to find a good friend or a good person to turn to. But if you had an online app then, you know, you could be able to turn your phone on and have a chat or something. It would be helpful.

Patient 13

Patient participants expressed interest in discussion boards, group chats, or video meetups that would allow individuals to come together and share their experiences and ideas. These could be initiated by program staff, with a defined topic, by users “throwing out a question,” or a web-based hangout. Participants and providers also conveyed concern about the potential for unhealthy interactions, in which people might be taken advantage of or encouraged to drink. They suggested mitigation strategies such as having a moderator to look out for individuals in crisis, misinformation, or problematic interpersonal or group dynamics, as well as post and enforce rules of conduct for the app.

Of the 24 patients we spoke with, only 1 was skeptical of the value of peer support in any form, believing that individual motivation and discipline are paramount:

People that need help need to keep up with their schedules and go to their treatments and try to—people [who use] alcohol, it’s their problem. They can’t do it from other—people can’t tell them what to do. They have to do it themselves.

Patient 3

### Clinician Support

Patient participants’ views varied regarding contact with counselors or other behavioral health providers within the app. Some of the participants considered counselor engagement a beneficial feature, particularly for providing trustworthy information and for immediate referrals or brief interventions for individuals in crisis. Others felt that having counselors available if needed—but not interjecting themselves—would be useful. Still others considered that having counselors as part of the app would discourage user engagement.

### Content

Patient participants identified a number of content types that should be included in the app. These included educational information about alcohol (eg, its effects on the body, symptoms of withdrawal, and health benefits of quitting) and culturally specific content (eg, audio or video stories from elders and Alaska Native music). We also heard that inspirational talks from people in recovery—“testimonials” in the language of some participants—would be valuable. Practical content aimed at helping users to enhance their self-management skills was also important. Participants recommended specific topics such as “how to avoid [risky] situations” and “how to ask for help.”

Comprehensive, up-to-date information about local resources and events was another desired content category. Patient participants said that the app should harness smartphones’ capacity to use geographic information system mapping to provide current, hyperlocal information to meet users’ needs. Suggestions from patients and providers included information about treatment services, Alcoholics Anonymous meetings, and crisis resources but were not limited to alcohol-related resources or events; for example, the participants also mentioned information about local cultural activities (eg, dancing and beading workshops), social events not involving alcohol (eg, family movie nights and hiking meetups), and listings such as 24-hour coffee shops (“Somewhere you can go, and be around people, even if you don’t know those people, and you can sip coffee and eat pie until the craving and the urge goes away, or until that meeting...or until your sponsor gets off work and can pick you up” [Provider 4]).

### Cultural Adaptation

Patient participants’ suggestions regarding the app’s look and feel included both culturally specific elements as well as more general Alaska-related themes. In the cultural domain, participants recommended including photographs or other images representing Alaska Native culture, such as beadwork, drums, dancing, or subsistence-related activities such as berry picking or fishing; for example, a patient commented as follows:

It would be good if the [app] wallpaper changed with the season and had in the background what [subsistence foods were] in season then. So in spring we get wild celery—what we call Yaan.eit—or, you know, the beach greens, and then, you know, the different...devil’s club and the goose tongue and, you know, cockles and gumboots and the different salmon; have that change with the seasons so you get that subtle reminder, “This is a time when you get this.” And it can be from all over the state, what is in season that week.

Patient 16

Participants also suggested tailoring color schemes, visual images, and perhaps some language to the user’s specific cultural group (eg, providing a daily greeting in Dena’ina Athabascan to those who identify as Dena’ina when they set up their user profile). Views regarding the acceptability of “pan-Indian” imagery were mixed. Some participants warned against the use of visual elements that are often used as generic “Native” design elements (eg, feathers and medicine wheel imagery). Others felt that such cultural references are shared by Indigenous peoples of North America and would be appropriate for use in an app intended to be used by ANAI people across the state, and perhaps even beyond Alaska. Additional suggestions included connecting to land and place by making use of nature scenes such as imagery of mountains, water, tundra, and northern plants (eg, fireweed and salmonberries) and animals (eg, bears and salmon).

Patients identified spirituality as an important contributor to sobriety, although views differed about whether or how to include spiritual or religious elements in the app. Some participants felt that such content was critical, as in a comment that the app should “respect people that love God.” Others voiced concern that the app should not appear to favor or “push” particular belief systems. Striking a balance between recognizing the importance of spirituality while allowing room for difference was critical, as exemplified in this quotation:

[H]aving anything to do with a specific religion [in the app] would be rude to everyone else who’s not part of that specific religion. I think it’s important for people who believe in a Creator or whatever version of spirituality that they have...that’s a healthy way of being part of the community, and it does help in recovery, but it’s really important to address those questions in a very religious-neutral way....We’re a very diverse [Indigenous] community [in the region], and that is one way that you’ll shut people off completely, if you go the wrong direction.

Patient 16

There was a wide range of views in our sample. Some of the patients said that they would not use an app that included only Christian references or themes, whereas some stated that they would find “pan-Indian” representations of spirituality inappropriate and disrespectful. Others considered spirituality too personal to be adequately addressed in an eHealth context. Participants cautioned that if the spiritual component were offered in a way that felt inauthentic, it could increase shame and stigma among would-be users and discourage, rather than support, their use of the app.

### Tone

A recurring theme throughout the interviews with patients and providers alike was the need to attend to tone: the app should avoid stigmatizing alcohol misuse, “talking down” to users, or taking a moralistic stance. Patient participants wanted the app to feel that it was “for us,” meaning for ANAI people. As noted previously, they preferred that the app include recognizable visual connections to the culture and the land and have neither a “corporate” nor a clinical feel. Patient participants also emphasized that the app should be free to users and should not include in-app advertising, purchases, or marketing pitches.

## Discussion

### Principal Findings

In this study, both ANAI patients and their providers endorsed a culturally adapted smartphone app for sobriety support. Participants valued the inclusion of self-management tools that would generate positive feedback and in-app recognition for achieving goals, as well as connections to locally available treatment options. They were also interested in material concerning spiritual and religious beliefs; ANAI cultural content; social services, such as those providing help with job searching or transportation; and sober meetups as well as dancing, crafting, and other local events that would support sobriety and cultural identity. Patients also strongly endorsed the option to participate in peer support conversations without disclosing their identity. Those who thought clinician support should be included said that such engagement should be offered in a nondirective way (unless a person was in acute crisis).

Prior studies have demonstrated the effectiveness of peer and social support in a variety of sobriety interventions [45-49]. Individual-focused interventions that have proven successful in the general population, such as cognitive behavioral therapy, may be less suitable for individuals from more collectivist cultures, including ANAI people. Given that feeling disconnected—from others and from their culture—is a driver of alcohol misuse among ANAI people, peer and other social support is likely a key component of self-management interventions for this group [9].

Our findings regarding participants’ desire for ANAI cultural content and peer support to be included in the app are consistent with research in other Indigenous communities that demonstrates the value of cultural connections in promoting and supporting sobriety and behavioral health [50-55]. Enculturation and a sense of belonging have been identified as protective factors in several prior studies; conversely, their lack has been associated with increased risk for substance misuse and suicidality [9,55-60]. In particular, the harvest, preparation, preservation, and sharing of traditional subsistence foods (eg, berries, salmon, and moose) provide physical, social, and spiritual sustenance to ANAI people [61,62]. Our data suggest that incorporating information or activities involving these foods and perhaps identifying more easily accessible alternatives (eg, store-bought approximations or plants that can be foraged in urban areas) could make an off-the-shelf app more appealing and meaningful for ANAI users. We believe that this study’s results regarding ANAI patients’ views of spiritual and religious content in an app for alcohol self-management are new to the literature.

Prior research has demonstrated that contingency management strategies, including prizes and cultural activities, have high acceptability among younger AI adults, who are also more frequent and proficient users of mobile technologies [32,60]. Our participants’ endorsement of incentives for app use and goal achievement—even in the form of in-app “rewards” such as brief, celebratory animations—is in line with these results. In the eHealth context and in light of the ubiquity of social media, finding ways of providing real-time reinforcements that users find motivating is an important area of study.

Participants in this study articulated a holistic view of well-being that included physical, psychological, social, spiritual, and cultural elements, all of which they wanted the app to address. These findings are in line with prior work identifying structural and ecological factors, such as stable housing and transportation, as potential barriers or facilitators in the context of sobriety and care seeking for alcohol misuse [9]. Study participants’ emphasis on choosing whether and how to use the app, not being talked down to by the app or engaged clinicians, and the ability to remain anonymous echoes the findings of studies that have highlighted the importance of self-determination and norms of noninterference among ANAI people seeking to change health-related behaviors [63-66].

This formative work highlights the importance of cultural adaptation as well as the challenges involved in tailoring a smartphone app for use by a culturally heterogeneous ANAI population; for example, although patient participants spoke of the importance of including resources and content related to spirituality, opinions about what content individuals would find supportive—for example, references to Christianity or Iñupiat spiritual beliefs—were highly variable. Moreover, some of the participants said that an inauthentic, overly prescriptive, or “pan-Indian” approach would dissuade them from using the app. Our results, as well as prior research, support the value of spiritual and religious practices as a protective factor against alcohol misuse and an aid to recovery among ANAI people [57,67-72]. Determining the feasibility of offering relevant options for potential ANAI users, together with exploring mechanisms to enable users to configure this feature themselves, will be a focus of the next phase of research.

### Limitations

This study was conducted in a single health care system serving ANAI people in the south-central region of Alaska. Our sample is unlikely to capture the views of the entire ANAI community in the south-central region, which includes people from many different cultural groups. Despite our targeted efforts to recruit rural patients in the region, fewer than a third (7/24, 29%) of the patient participants lived outside the Anchorage metropolitan area. These results may not represent the perspectives of ANAI people in other regions or of individuals who are unwilling to talk about alcohol misuse or associated problems or share their opinions in a research setting.

### Conclusions

In this study in south-central Alaska, ANAI patients and their providers were receptive to the idea of a smartphone app for sobriety support and offered concrete suggestions for culturally appropriate adaptations. These included look-and-feel changes as well as specific culturally grounded content and features. Although this finding may or may not generalize to other contexts, we recommend that similar needs assessments not constrain participants’ feedback to surface-level adaptations. The degree to which a given off-the-shelf solution can accommodate deeper adaptations may be an important part of the tool and vendor selection process.

Participants expressed the view that an app could fill a gap in support for rural-dwelling ANAI people having difficulty accessing alcohol treatment services as well as those with limited social support and those who may not wish to seek formal treatment. Smartphone apps may have particular value in settings such as this one in which geographic remoteness and the privacy concerns that can arise in small communities may make in-person peer support unattractive to intended users or difficult to implement. These findings may be of use to other tribal health organizations considering adapting commercially available eHealth interventions to help ANAI people manage their alcohol use.

## Abbreviations

ANAI: Alaska Native and American Indian
SCF: Southcentral Foundation
SIP-2R: Short Inventory of Problems, revised

## Data Availability

The data set presented in this paper is not publicly available. Data access is contingent upon tribal review and approval. Contact author AFC for information about the tribal review process.
